# Trypsin Genes Are Regulated through the miRNA Bantam and Associated with Drug Sensitivity in the Sea Louse *Caligus rogercresseyi*

**DOI:** 10.3390/ncrna7040076

**Published:** 2021-12-03

**Authors:** Gustavo Núñez-Acuña, Valentina Valenzuela-Muñoz, Crisleri Carrera-Naipil, Constanza Sáez-Vera, Bárbara P. Benavente, Diego Valenzuela-Miranda, Cristian Gallardo-Escárate

**Affiliations:** 1 Interdisciplinary Center for Aquaculture Research (INCAR), University of Concepción, Concepción P.O. Box 160-C, Chile; valevalenzuela@udec.cl (V.V.-M.); crislericarrera@gmail.com (C.C.-N.); saezvera.c@gmail.com (C.S.-V.); bbenavente@udec.cl (B.P.B.); divalenzuela@udec.cl (D.V.-M.); crisgallardo@udec.cl (C.G.-E.); 2 Laboratory of Biotechnology and Aquatic Genomics, Department of Oceanography, University of Concepción, Concepción P.O. Box 160-C, Chile

**Keywords:** *bantam*, *trypsin*, drug sensitivity, pharmacological resistance, sea lice, *Caligus rogecresseyi*

## Abstract

The role of *trypsin* genes in pharmacological sensitivity has been described in numerous arthropod species, including the sea louse *Caligus rogercresseyi*. This ectoparasite species is mainly controlled by xenobiotic drugs in Atlantic salmon farming. However, the post-transcriptional regulation of *trypsin* genes and the molecular components involved in drug response remain unclear. In particular, the miRNA *bantam* family has previously been associated with drug response in arthropods and is also found in *C. rogercresseyi*, showing a high diversity of isomiRs. This study aimed to uncover molecular interactions among *trypsin* genes and *bantam* miRNAs in the sea louse *C. rogercresseyi* in response to delousing drugs. Herein, putative mRNA/miRNA sequences were identified and localized in the *C. rogercresseyi* genome through genome mapping and blast analyses. Expression analyses were obtained from the mRNA transcriptome and small-RNA libraries from groups with differential sensitivity to three drugs used as anti-sea lice agents: azamethiphos, deltamethrin, and cypermethrin. The validation was conducted by qPCR analyses and luciferase assay of selected *bantam* and *trypsin* genes identified from in silico transcript prediction. A total of 60 *trypsin* genes were identified in the *C. rogercresseyi* genome, and 39 *bantam* miRNAs were differentially expressed in response to drug exposure. Notably, expression analyses and correlation among values obtained from *trypsin* and *bantam* revealed an opposite trend and potential binding sites with significant ΔG values. The luciferase assay showed a reduction of around 50% in the expression levels of the *trypsin 2-like* gene, which could imply that this gene is a potential target for *bantam*. The role of *trypsin* genes and *bantam* miRNAs in the pharmacological sensitivity of sea lice and the use of miRNAs as potential markers in these parasites are discussed in this study.

## 1. Introduction

The most relevant infectious disease caused by an ectoparasite species in the Chilean farmed salmon industry is Caligidosis [1]. The infective agent of this disease is the marine copepod *Caligus rogercresseyi*, which is widely distributed in salmon farms in Southern Chile [2,3]. The losses caused by Caligidosis to the salmon industry are in the order of hundreds of millions of US dollars per year [4,5]. The control for this disease mainly consists of drug treatments, including organophosphate, pyrethroid, and avermectin molecules [6,7]. However, the control by the use of these molecules has been threatened by the emergence of pharmacological resistance in *Lepeophtheirus salmonis* [8] and the reduced efficacy reported in *C. rogercresseyi* [9,10]. This evidence is supported by the identification of putative drug detoxification systems and excretion mechanisms found in sea lice species [11].

The molecular mechanisms behind pharmacological resistance are not yet wholly described in marine ectoparasites. In terrestrial arthropods, pharmacological resistance mechanisms involve mutations in target genes, drug detoxification and metabolism, and morphological variations [12,13,14]. In sea lice species, mutations in target genes for delousing drugs have been associated with resistant genotypes in *L. salmonis* widely distributed among the Northern Hemisphere coasts [15,16]. However, recent studies applying next-generation sequencing techniques have described diverse and complex molecular responses that suggest novel pharmacological resistance mechanisms in the sea louse *C. rogercresseyi* [11,17,18]. Among these genes, *trypsins* are a group of genes involved in drug detoxification and differentially expressed in *C. rogercresseyi* exposed to azamethiphos and deltamethrin drugs [19]. These genes are present in the secretory/excretory products (SEPs) in ectoparasites [20,21]. From *trypsins*, 44 different isoforms have been described in *C. rogercresseyi* [19]. In addition, two isoforms of *trypsin* genes showed strong transcriptional differences between susceptible and resistant lice strains to the pesticide deltamethrin. Notably, findings have suggested putative molecular interactions with non-coding genes [11].

The molecular mechanisms behind pharmacological resistance are not exclusive to coding genes. Non-coding RNAs, including miRNAs, have a role in drug exposure responses and pharmacological resistance in several species [22]. For example, in arthropods, miRNAs regulate the expression of genes related to drug detoxication, conferring pharmacological resistance to drugs such as chlorantraniliprole [23] and pyrethroids [24,25]. In the sea louse *C. rogercresseyi*, previous investigations identified the whole miRNome and its association with ontogeny development during its lifecycle [26]. One of the most abundant families of miRNAs is *bantam*, which is highly up-regulated in the infective developmental stage of this parasite (copepodid). A newer version of the *C. rogercresseyi* miRNome has just been published, and an association with resistant strains to three delousing drugs was found [27]. In this study, *bantam* was also described as a key miRNA family, putatively interacting with genes related to drug detoxication. The differentially expressed miRNAs in lice exposed to delousing were also putatively associated with long non-coding RNAs (lncRNAs). This class of non-coding RNAs has also been identified as having a potential role in the drug response [28]. 

The objective of this study was to identify, evaluate, and validate the putative molecular interactions among *trypsin* genes and *bantam* miRNAs in the sea louse *C. rogercresseyi* exposed to delousing drugs. These genes were selected as candidates based on previous evidence related to their abundance and role in drug responses of *trypsins* and *bantam*. Furthermore, previous research in other arthropod species has described potential interactions of novel miRNAs—bantam included—with serine protease genes [29]. However, this is the first study to explore the molecular interplay between miRNA *bantam* and mRNA *trypsin* in sea lice, and their putative roles in drug response and pharmacological resistance.

## 2. Results

### 2.1. Identification of Trypsin Genes and Bantam miRNAs in the C. rogercresseyi Genome

**Figure 1:**
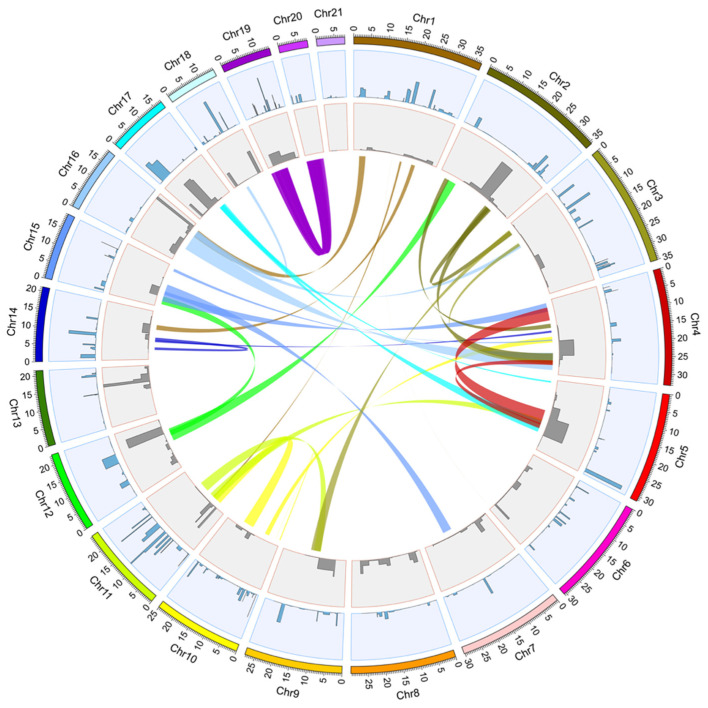
Identification of trypsin and bantam genes in the *C. rogercresseyi* genome through CIRCOS plot. Chr1–Chr21 colored segments correspond to the complete sequence of the chromosomes of the sea louse genome using a chromosome unit value of 100,000 bp. The first track (light blue) corresponds to a histogram plot of reads mapped in genes annotated as trypsin, trypsin-like, and chymotrypsins for this species. The second track (gray) shows the read mapping for annotated bantam miRNAs in the sea louse genome. Links correspond to syntenic blocks in the *C. rogercresseyi* genome obtained from blastN analyses of 10,000 bp segments throughout the whole genome.

A total of 60 trypsin genes were mapped and annotated in the genome of *C. rogercresseyi*. These genes were distributed in the 21 chromosomes, but the mapped reads were mainly located at chromosomes 3, 1, 10, and 11 (Figure 1). The gene lengths were highly divergent among the trypsins, where the shortest was a chymotrypsin-like elastase family member with 315 bp located in chromosome 5, and the longest was trypsin-1 with 44,817 bp located in chromosome 13 (Table 1). Thus, the average length of trypsin genes was 4518 bp. Genes with multiple transcripts were also abundant, ranging from 1 to 7 transcripts per gene. The shortest transcript coding for a trypsin mRNA was 240 nt in length, and the most extended identified transcript had a length of 4522 nt. Thus, the average length for transcripts coding for trypsins was 1042 nt. The direction of transcripts was inferred in 43 trypsin genes, where 22 were identified in the positive (+) and 21 in the negative strand (−).

On the other hand, 155 different mappings for miRNAs from reads assembled and annotated as members of the bantam family were obtained from the sea louse genome (Figure 1). These mappings were ubiquitously distributed in all the chromosomes, with more bantam in chromosomes 7 and 12. However, most of the mapped reads were located at chromosomes 13 and 16, followed by chromosome 6, which related to higher expression levels (Supplementary Table S1).

### 2.2. Expression Analyses of Bantam and Trypsin Genes

**Figure 2:**
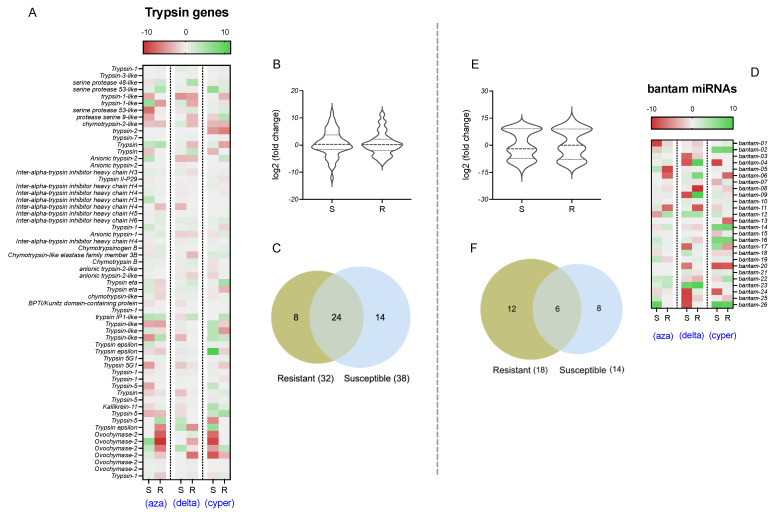
In silico expression analyses for trypsin (**A**–**C**) and bantam (**D**–**F**) in *C. rogercresseyi* exposed to delousing drugs. (**A**,**D**) Heatmaps for fold change values calculated on exposed samples vs. control for differentially expressed trypsin genes and bantam miRNAs, respectively. Significant expression changes were considered if the fold change > |4| and *p*-value > 0.01. (**B**,**E**) Violin plots of the mean expression values of all the differentially expressed trypsin genes and bantam miRNAs, respectively, separated by susceptible and resistant populations to delousing drugs. (**C**,**F**) Venn diagrams for trypsin genes and bantam miRNAs evaluated in (**B**,**E**) plots.

Expression differences were found in trypsin genes and bantam miRNAs in samples exposed to delousing drugs from susceptible and resistant populations (Figure 2). Expression differences were found in susceptible vs. resistant populations exposed to the same drug. From the 60 trypsin genes, there were expression differences according to resistant/susceptible populations in 46 genes (Figure 2B,C). Overall, there was a reduction in the mean expression of trypsin genes in susceptible populations than in resistant lice populations. There were 38 trypsin genes with expression patterns associated with the susceptible populations, including 14 that were differentially expressed only in these populations. Thirty-two trypsins had expression patterns associated with resistant populations, including eight that were only differentially expressed in these populations. In addition, 24 trypsins were differentially expressed in lice exposed to drugs obtained from resistant and susceptible populations.

Regarding bantam miRNAs expression, a lower number of transcripts showed differential expression changes in sea lice, with a total of 26 bantams with a significant expression associated with either susceptible or resistant populations (Figure 2E,F). Bantam expression was also slightly reduced in susceptible populations compared to resistant populations for the three drugs. A total of 26 bantams had significant expression differences in lice exposed to drugs from the resistant populations, whereas 12 had expression patterns associated only with resistant populations. In contrast, 14 bantams’ expression was observed in susceptible populations, including exclusive expression patterns in 8 miRNAs. Additionally, six bantam miRNAs were differentially expressed in lice exposed to drugs in susceptible and resistant populations. When trypsin and bantam expression levels were observed in heatmaps to find differences in single transcripts (Figure 2A,D), more contrasting differences in experimental groups (different drugs, or susceptible vs. resistant population comparisons) were found in bantam miRNAs than in trypsin genes. In these heatmaps, fold change values for bantam expression were closer to the limits of the scale than trypsin expression values.

### 2.3. Expression Value Correlations

**Figure 3:**
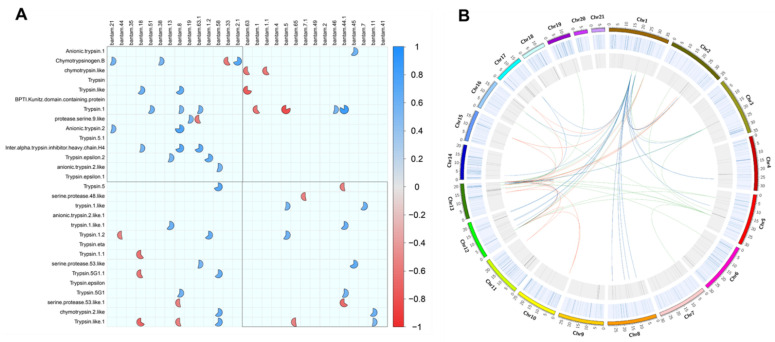
Correlation analysis of expression pairs between trypsin and bantam TPM values. (**A**) Corrplot analyses were conducted on differentially expressed trypsins and bantams (fold change > |4| and *p*-value > 0.01) among all of the data, including the exposure to the three delousing drugs. Only Pearson’s correlation values that were significant (*p*-value > 0.01) are shown in the plot. Red pies correspond to significant negative correlations and blue pies to significant positive correlations. The completeness of the pies corresponds to the correlation level, where pies closer to a circular shape correspond to values more proximal to |1| in Pearson’s calculation. (**B**) Correlations linked to chromosome positions in the salmon louse genome. The two tracks correspond to heatmaps of reads mapped in genes annotated as trypsin, trypsin-like, and chymotrypsins (light blue track) and to heatmaps of annotated bantam miRNAs (gray track) in the sea louse genome. Colored links in the center of the plot correspond to significant negative correlations (r < −0.95; *p* < 0.01) for the expression pairs between trypsin genes and bantam miRNAs for a specific delousing drug exposure, including resistant and susceptible populations. Blue links correspond to exposure to azamethiphos, green to deltamethrin, and red to cypermethrin.

From the 32 trypsin genes and 26 bantam miRNAs with expression patterns associated with resistant/susceptible populations, significant correlations in these expression profiles were obtained through Pearson’s calculations and the corrplot package. Combining the data obtained from the exposure for each of the three delousing drugs, a total of 53 significant correlations (|r| < 0.9; *p*-value < 0.05) were found in the expression of 23 different trypsin genes and 24 different bantam miRNAs (Figure 3). A total of 17 significant negative correlations (r < −0.9; *p*-value < 0.05) were obtained in these data. However, these data combined expression values obtained from different populations and exposure to three different drugs; therefore, some significant correlations could be missed. Using the expression data separated by each delousing drug, more significant correlations were obtained between trypsins and bantams and were associated with the exposure to each specific drug (see links in the CIRCOS plot in Figure 3). In the expression patterns of sea lice exposed to azamethiphos from both susceptible and resistant populations, 23 significant negative correlations were found (see blue links in Figure 3); 19 were found in samples exposed to deltamethrin (see green links in Figure 3); and 24 were found in lice exposed to cypermethrin (see red links in Figure 3). Even though trypsin genes and bantam miRNAs are distributed in various chromosomes in the sea lice genome, these correlations were concentrated in a few chromosomes and delousing drugs. A total of 82.61% of negative correlations in populations exposed to azamethiphos were associated with trypsins found in chromosome 1 only, and the remaining 16.67% were associated with trypsins found in chromosome 4. In populations exposed to deltamethrin, negative correlations occurred in trypsins from only three chromosomes: 47.37% in chromosome 5, and 26.32% in chromosomes 1 and 13. In populations exposed to cypermethrin, most of the significant correlations were found in only two chromosomes: 45.83% in chromosome 13 and 37.50% in chromosome 1, and the remaining correlations in trypsins were from chromosomes 10 and 11, with 8.33% each.

### 2.4. Expression Validation through qPCR

**Figure 4:**
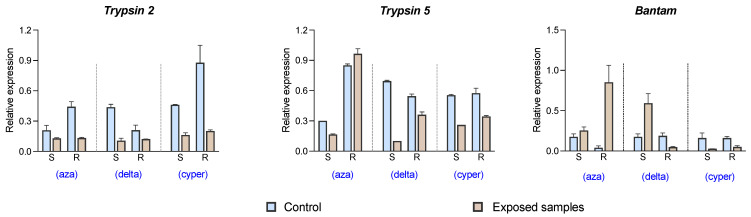
Validation of gene expression levels of bantam, trypsin-2, and trypsin-5 by qPCR analyses. Relative expression is calculated by ΔΔC_T_ values after normalization with the corresponding endogenous control (mir-2796 for bantam expression and b-tubulin for both trypsin genes). Light blue columns correspond to control sea louse samples, and light brown to samples exposed to the respective drug: “aza” = azamethiphos; “delta” = deltamethrin; “cyper” = cypermethrin. Error bars correspond to standard deviation. S: susceptible population; R: resistant population.

Two trypsin genes (trypsins 2 and 5) and one bantam sequence were used to validate in silico expression data by qPCR. These three selected genes showed differential expression values in resistant and susceptible populations for the three delousing drugs (Figure 4). In the qPCR analysis results, bantam showed an increase in expression levels in the resistant population to azamethiphos after drug exposure and a reduction in expression in the susceptible population to deltamethrin after drug exposure. Significant variations between control and exposed samples were found in most of the experimental groups. The most significant variation in gene expression for trypsin 5 had an opposite trend with respect to bantam expression, where sea lice from the susceptible population reduced the expression levels after drug exposure. In contrast, the most relevant expression changes in the trypsin 2 gene were found in the samples from the populations exposed to cypermethrin. This latter gene presented an opposite trend to the expression of bantam miRNA, showing a reduction in expression in the exposed samples to azamethiphos and deltamethrin.

### 2.5. Validation of Trypsin Binding Site for Bantam through Luciferase Assay

**Figure 5:**
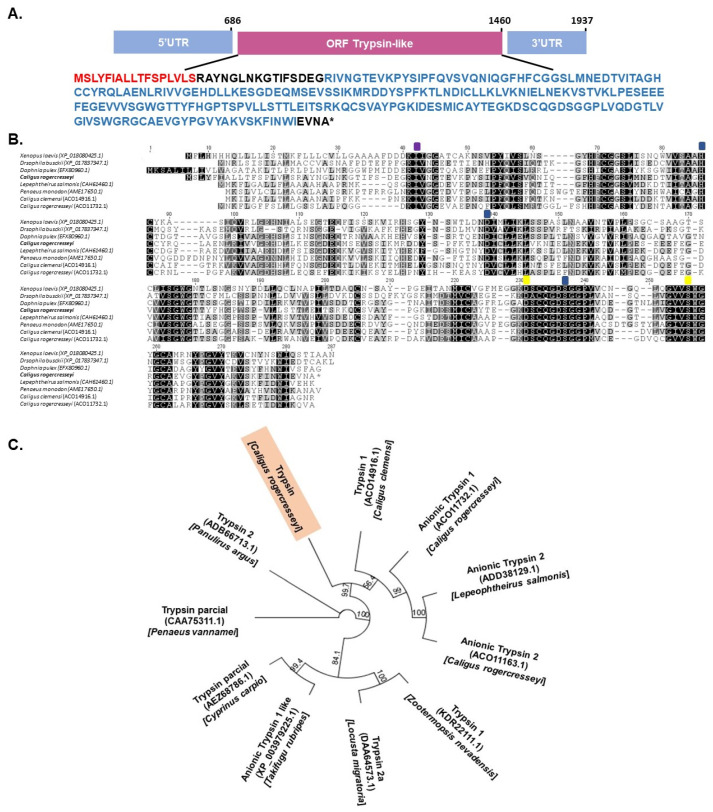
Characterization of trypsin-2 gene in the sea louse *C. rogercresseyi*. (**A**) Schematic representation of full cDNA of trypsin-2 sequence. In red: peptide signal; in blue: trypsin-like serine protease domain. (**B**) Multiple alignments of trypsin-2 genes. Purple square represents the cleavage site, and yellow squares the substrate union site. The blue squares indicate the active site. (**C**) Phylogenetic analysis of trypsin-2 gene. The tree was constructed by neighbor joining methods, with 1000 bootstrap replicates.

Due to the opposite trends in the expression values from trypsin 2-like and bantam, trypsin 2-like was selected as a candidate for identifying binding sites for this miRNA and for validation through a luciferase assay. The whole transcript sequence used for trypsin 2 had a total length of 1937 nt, with an ORF region of 774 nt coding for 257 amino acids. The 5′-UTR and 3′-UTR regions were 686 nt and 477 nt, respectively (Figure 5A). The structural analysis of the deduced protein sequence showed a serine protease domain and a putative endopeptidase activity site (Figure 5B). Multiple alignments with trypsin proteins described for other arthropods showed a high conservation level (Figure 5B). This trypsin from *C. rogercresseyi* showed 39.47% and 47.86% identity with trypsin proteins described for *Lepeophtheirus salmonis* and *Caligus clemensi*, respectively. Moreover, the phylogenetic analysis of trypsin 2 from *C. rogercresseyi* showed that this gene is in the clade for trypsins described in *C. clemensi* and two anionic trypsins previously described for *C. rogercresseyi* (Figure 5C).

**Figure 6:**
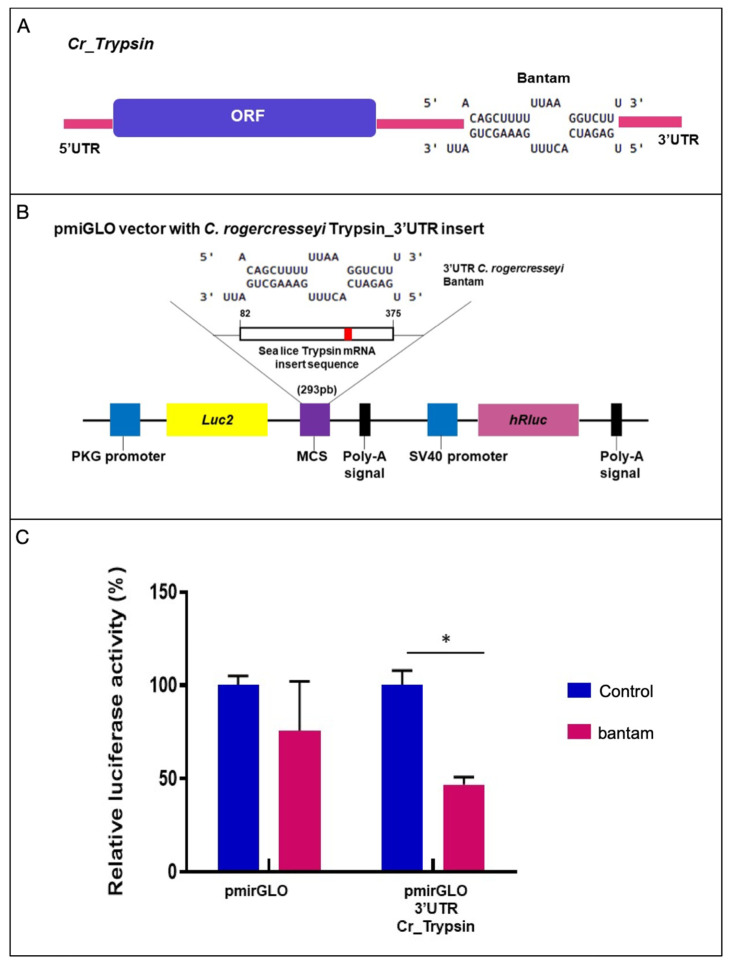
Validation of trypsin-2 target site for bantam miRNA. (**A**) The predicted binding site for bantam in 3′ UTR of trypsin-2 gene. (**B**) Schematic representation of pmirGLO-3′UTR trypsin vector construction. (**C**) Luciferase assay showing luminescence activity after exposure of pmirGLO-3′UTR trypsin vector to bantam mimic sequence. The asterisk indicates significant differences (*p* < 0.001). Control samples correspond to the vector without the trypsin-2 gene insert.

A binding site for bantam was identified from the target prediction analysis on the trypsin 2-like 3′-UTR region. The binding sequence corresponded to 5-ACAGCTTTTTTAAGGTCTT-3, located between nucleotides 1016 and 1033 of the 3′-UTR (Figure 6A). The hybridization energy (ΔG) was estimated at −20.7 kcal/mol. The target sequence was cloned in the pmiGLO vector for functional validation and transfected with a bantam mimic in Hela cells (Figure 6B). After 48 h, the luciferase activity was reduced by 50% in the group transfected with the bantam mimic (Figure 6C). This result confirms the binding site of bantam as the trypsin 2-like 3′-UTR, suggesting a regulatory function of sea louse bantam miRNA in trypsin expression.

## 3. Discussion

The impact of the sea lice species *C. rogercresseyi* and *L. salmonis* in the salmon farming industry represents one of the major concerns in the development of sustainable aquaculture. The reduction in the efficacy of therapeutic methods by anti-parasite drugs has motivated research focused on the emergence of pharmacological resistance through different perspectives. One described approach is identifying single mutations in the genes encoding for the target proteins of delousing drugs. In *L. salmonis,* targeted mutations in target genes have been associated with resistant populations [15], allowing the evaluation of the distribution of resistant genotypes to delousing drugs [16]. Regarding *C. rogercresseyi,* SNP polymorphisms have been described in the target gene for azamethiphos, with implications in the drug–protein active binding site [30]. However, these markers have not been described yet in resistant populations for this species. Another strategy in *C. rogercresseyi* is to identify several genes and pathways related to drug response through next-generation sequencing and RNA-seq analyses [11]. Thus, the characterization of resistant genotypes has been enhanced by describing molecular functions that were not evaluated at first for assessing pharmacological resistance in the sea louse. Transcriptome analyses have also allowed the description of novel non-coding RNAs (long non-coding RNAs and miRNAs) associated with delousing drug response in *C. rogercresseyi*, expanding the knowledge to other overlooked genomic regions [11,28]. Nonetheless, the characterization of the complete genome of *C. rogercresseyi* at the chromosome scale [31] could reshape the understanding of pharmacological resistance and delousing drugs in this species.

Indeed, this is the first time that miRNA–gene interactions have been described combining co-expression and chromosome localization data to characterize the delousing drug response in sea lice species. Through the circular representation of the genome, the *bantam* and *trypsin* gene expression uncovered the potential interaction regions within a single chromosome or from regions present at different chromosomes. These interactions may be involved in the expression regulation of genes with a critical function in delousing drug response and the emergence of pharmacological resistance. Thus, the next step is to elucidate whether these *bantam–trypsin* interaction regions, particularly those involving two different chromosomes, are associated with genomic regions that could be interacting due to chromatin/chromosome organization in the cell (e.g., topologically associating domains, TAD).

Pharmacological resistance mechanisms are not only limited to punctual mutations in target genes for specific drugs. Complex and diverse gene pathways are activated or inhibited after exposure to delousing drugs [11,17,18,28,32]. One of the commonly described mechanisms present in arthropod species, and also suggested for sea lice, is metabolic resistance, whose mechanism involves drug detoxication systems including genes involved in the absorption, distribution, metabolism, and excretion of drugs [33]. Our study was focused on the family of *trypsin* genes, which are serine proteases involved in proteolysis but possessing a catalytic triad in their structure, similar to those found in carboxylesterases and other genes involved in drug detoxication [34]. In addition, the role of *trypsin* genes in insecticide resistance has been suggested in a previous study through drug degradation [35]. In *C. rogercresseyi*, 51 putative *trypsin* genes (44 *trypsin-like* and 7 *chymotrypsin-like* genes) were discovered from transcriptome databases, whose SNP markers and expression patterns suggested a role in the response of sea lice to deltamethrin [19]. This study expands the knowledge by identifying 60 *trypsins* and *chymotrypsins* and describing expression profiles in sea lice exposed to azamethiphos and cypermethrin. Another significant contribution is the expression patterns described in sea lice populations with contrasting resistance/susceptibility to the three drugs used in this study. The expression patterns associated with susceptible or resistant populations were localized at genomic regions where the *trypsin* gene was identified, providing novel information such as the finding of spliced variants for these genes. The next step is to characterize spliced variants associated with pharmacological resistance in this species. Interestingly, most of the *trypsins* identified in this study were differentially expressed after drug exposure and according to resistant or susceptible populations (46 out of 60 genes), supporting the hypothesis of the role of *trypsins* in delousing drug response and expanding this to azamethiphos, deltamethrin, and cypermethrin. These results also support the hypothesis related to considering *trypsin* genes as biomarkers for delousing drug susceptibility, which was suggested for *C. rogercresseyi* in novel bioassays using azamethiphos [36], especially *trypsin-2* and *trypsin-5,* which were also validated by qPCR. In the qPCR validation of these two genes, the basal expression levels were also variable in control samples for both S/R populations (Figure 4). This is the reason to evaluate these genes as biomarkers not only considering unexposed samples but also exposed lice to drugs, and then to evaluate the differential expression of control vs. exposed samples in each population. The variations in the basal expression of these genes may be attributable to the different roles that *trypsins* could play in marine species, which can be triggered, for example, by environmental conditions in seawater [37]. Moreover, variation in control samples was also found in a previous study in *C. rogercresseyi* [19].

The *bantam* miRNAs comprise a broad family described exclusively in arthropods, identified for the first time in *Drosophila* [38], with roles in tissular growth regulation [39], cell proliferation [40], and other biological processes mainly studied in *Drosophila* spp. [41,42,43]. This family was identified along with the description of the first miRNome for *C. rogercresseyi*, including the expression profiles of miRNAs in different developmental stages [26]. Around 200 isoforms of *bantam* (isomiRs) were found in the first characterization of the *C. rogecrresseyi* miRNome, and this was the most up-regulated family of miRNAs in the infective developmental stage of these parasites. Furthermore, *bantam* miRNAs had potential interactions with genes related to drug detoxication, such as the enzymes *GST* and *Cytp450* and the transporter protein genes *ABC* and *SLC* [27]. These facts have led us to deeply study the *bantam* family as a candidate for delousing drug response in *C. rogercresseyi*. The first interesting point is that we only mapped 155 *miRNAs* on the sea lice genome, although more *bantam* sequences were found in transcriptome databases. This could be related to chromosome regions where the algorithm could not differentiate two very proximal *bantam* sequences in the genome due to the high conservation in the small sequences of these miRNAs. The next step to further describe *bantam* in the *C. rogercresseyi* genome is to evaluate the presence of spliced reads or variants corresponding to *bantam* sequences in the sea louse genome. Other interesting points for *bantam* expression patterns associated with delousing drugs compared with *trypsins* are the lower number of miRNAs with differential expression patterns from the total *bantams* identified, and the higher differences in fold change values. However, only 25% of the identified *bantam* miRNAs were differentially expressed, probably due to the diverse roles that these non-coding RNAs could play in the cell. Therefore, we used the 36 differentially expressed *bantam* miRNAs for further analyses, as these could be involved in delousing drug response in *C. rogercresseyi*.

Putative interactions between *trypsin* as the target gene and *bantam* miRNAs have been suggested in previous studies. In the sea louse, *C. rogercresseyi*, a potential target site was predicted in a *trypsin-3* gene for two *bantam* isoforms [26]. In that study, predicted target sites were found for *bantam* in the *serine protease* and *chymotrypsin-2* genes. A putative target site for *bantam* in another *chymotrypsin* gene was found in the salmon louse species in the Northern Hemisphere, *L. salmonis* [44]. In other arthropods, such as *Helicoverpa armigera*, predicted target sites have been found for *trypsin* and *chymotrypsin* genes, but also for other miRNAs not belonging to the *bantam* family [29,45]. None of these studies have focused on regulating the *trypsin* gene by *bantam* miRNA in the context of therapeutic drugs or pharmacological resistance. However, the significant contribution of the present study can be summarized in two aspects with respect to *trypsin* and *bantam* interactions: correlation of gene expression levels at spatial regions in the chromosomes of *C. rogercresseyi*, and validation of a *trypsin–bantam* target site by the luciferase assay.

Regarding correlation analyses, various correlations were found combining the data from exposure to the three drugs. However, the most interesting result is the negative correlations of gene expression for resistant and susceptible populations separated by drug and found in chromosome regions. Conversely to what was expected, significant correlations were found in different *bantam* and *trypsin* genes, even for deltamethrin and cypermethrin drugs which have similar structures since both are pyrethroid molecules [46]. These results could lead to identifying specific genomic regions involved in the resistance mechanisms for specific drugs. However, there are still chromosome regions of particular interest due to the greater number of *bantam–trypsin* interactions found, such as chromosomes 1, 12, and 13. On the other hand, regarding the luciferase assay, it was possible to validate the *trypsin 2-like* target site for *bantam,* which was a technique previously validated for other miRNA–mRNA binding sites [47]. Thus, the regulation of the expression of *trypsin-2* by *bantam* adds more value to the interest for this gene, which has been suggested as a biomarker for drug sensitivity in *C. rogercresseyi* [36]. Future investigation will be conducted to evaluate gene–miRNA interaction through in vivo assays, and to explore if *bantam* could reduce the expression of the *trypsin-2* gene, altering the molecular response of the sea louse *C. rogercresseyi* to delousing drugs.

## 4. Materials and Methods

### 4.1. Identification of Trypsin Genes in Sea Louse Genome

The transcriptome database of the sea louse *Caligus rogercresseyi* exposed to three delousing drugs (azamethiphos, deltamethrin, and cypermethrin) was used to identify *trypsin* genes (transcriptomes in SRA database, accession number PRJNA559846). These samples corresponded to resistant and susceptible sea lice populations for each drug [11]. All the reads were mapped to the full genome of this species (Genome GenBank database accession number PRJNA551027) [31], using the CLC Genomic Workbench software (version 20.0, Qiagen Bioinformatics). In this software, the Large Gap Read Mapping tool was applied to map the trimmed reads from the transcriptomes on the 21 chromosomes of the reference genome using the following parameters: maximum number of hits for a segment = 10; maximum distance from seed = 50,000; match score = 1; mismatch cost = 2; insertion cost = 3; deletion cost = 3; length fraction = 0.8; similarity fraction = 0.8. The transcript discovery tool was used to predict genes using the following parameters: minimum length of ORF = 200; minimum unspliced reads = 5; gene merging distance = 50; minimum reads in gene = 10; minimum predicted gene length = 250. The obtained putative transcripts were blasted (blast-X) against a specific database from *trypsins* for arthropods obtained from non-redundant databases from GenBank and UniProt. Putative predicted *trypsin* genes that had *e*-values > 1 × 10^−5^ were considered for further analyses.

The identified *trypsin* genes were localized in the sea louse genome using the data from CLC Genomics software and by plotting them in a circular representation of the genome using the software CIRCOS [48]. Histograms of mapped reads per genomic position by each chromosome were plotted as a track into the circular visualization in this software. The karyotype of the *C. rogercresseyi* genome was used as a reference by defining each chromosome size, and the chromosome units were 100,000.

### 4.2. Identification of Bantam miRNAs in Sea Louse Genome

The small-RNA sequencing database from the same samples used in the previous section was used for these analyses (small-RNA Truseq libraries deposited in the same SRA project number PRJNA559846) [27]. The trimmed reads were mapped to the *C. rogercresseyi* genome in the CLC Genomics software using the following parameters: match score = 1; mismatch cost = 2; cost of insertions and deletions = linear gap cost and 3 for each; length fraction = 0.9; similarity fraction = 0.9. Mapped small-RNA reads were extracted by the “extract and count” tool in CLC Genomics, including ambiguous read and low-quality (>0.05) read filtering. Truseq sequencing adapters were also trimmed at this point, and all the reads were shorter than 15 nt and longer than 25 nt. Annotation of reads was conducted by merging counts and blast against miRbase (release 22.1), selecting species corresponding to arthropods, and allowing 2 maximum mismatches and 2 missing bases. Those sequences annotated as *bantam* were selected for further analyses from the list of putative miRNAs that passed all the cutoffs. The reads included in the individual assemblies that were annotated as “*bantam*” were extracted and mapped back to the genome of *C. rogercresseyi* to identify the localization of potential *bantam* mature sequences at the chromosome level. Putative *bantam* miRNAs were localized in the *C. rogercresseyi* genome by circular visualization in CIRCOS [48], following the same parameters used for *trypsin* and merging the plot in one analysis as a second track.

### 4.3. Expression Analyses of Trypsin and Bantam Genes

In silico expression analyses were conducted in the identified *trypsin* genes and *bantam* miRNAs in the CLC Genomic workbench. RNA-seq analyses were conducted using the samples exposed to different drugs and controls (GenBank project accession PRJNA559846), using *trypsin* as a reference, and miRNA-seq analyses were conducted using *bantam* as a reference. For both analyses, expression values were expressed in TPM (total reads per million base pairs) and were calculated establishing a mismatch = 2, insert and deletion costs = 2, and length and similarity fractions = 0.8. Hierarchical clustering was conducted on both *trypsin* and *bantam* expression values to visualize changes in TPM values in all the samples, clustering the data by Euclidean distances with a complete linkage. Statistical analyses were based on generalized linear model regressions, automatically calculated on fold change values in CLC Genomics, and lice that were not exposed to drugs were used as control samples. Differentially expressed *trypsins* or *bantams* were considered if |fold change| < 4 and *p*-value > 0.01. Statistical comparisons were also estimated comparing resistant and susceptible populations by each delousing drug.

### 4.4. Gene Expression Validation by qPCR

The same samples used in a previous study of sea lice exposed to delousing drugs were used to validate RNA-seq and miRNA-seq analyses [11]. From this set of samples, control lice (not exposed to any drug) and lice exposed at 100 ppb of azamethiphos, 3 ppb of deltamethrin, and 15 ppb of cypermethrin were selected for validation by qPCR analyses. These concentrations corresponded to the recommended doses to apply in delousing drug treatments in Chile. Samples used in this analysis included lice from the resistant and susceptible populations for each drug. RNA extraction was conducted in all the samples using the Trizol reagent method (Invitrogen^®^, Waltham, MA, USA), following the protocol provided by the manufacturer. Validation of *trypsin* gene transcription was conducted on 200 ng of extracted RNA, and cDNA synthesis was performed using the RevertAid H Minus First Strand cDNA Synthesis kit (ThermoFisher Scientific, Waltham, MA, USA). Primers and probes for the *trypsin 2* and *5* genes were used as in a previous study [36], including an endogenous control gene *b-tubulin* to perform a triplex TaqMan^®^ assay (Table 2; Life Technologies™, Carlsbad, CA, USA). The qPCR reactions consisted of 20 μL of total volume using the Kapa Probe fast qPCR Master Mix 2X kit (Kapa Biosystems, Wilmington, MA, USA) following the default protocol provided by the manufacturer. Thermal cycling conditions consisted of 95 °C for 3 min of initial denaturation, followed by 40 cycles of 95 °C for 3 s and 65 °C for 20 s, and the process was conducted in triplicates in a QuanStudio™ 3 system (Life Technologies™, Carlsbad, CA, USA).

Validation of *bantam* transcription levels was achieved by synthesizing cDNA from the same RNA samples using the kit miSCript II RT (Qiagen, Hilden, Germany), with an incubation reaction at 37 °C for 60 min and 5 min at 95 °C. Specific primers were designed for *bantam* miRNA and were used for amplification by qPCR using the miScript SYBR Green PCR kit (Qiagen, Germany) in a QuanStudio 3 System (Life Technologies™, Carlsbad, CA, USA). Thermal cycling conditions consisted of an initial denaturation and enzyme activation at 95 °C for 15 min, followed by 40 cycles of 94 °C for 15 s (denaturation), 55 °C for 30 s (annealing), and 70 °C for 45 s (extension). The *mir-2796* miRNA was used as an endogenous control for this reaction, as was previously validated for *C. rogercresseyi* exposed to delousing drugs [26]. For *trypsin* and *bantam* qPCR runs, the transcriptional activity was quantified using the ΔΔC_T_ comparative method previously described for gene expression analyses [49]. Statistical analyses from ΔΔC_T_ values corresponded to an application of a Kolmogorov test to evaluate the distribution of the obtained data, and then ordinary one-way ANOVA was used on data with a Gaussian distribution, or the Kruskal–Wallis test if that distribution was not achieved.

### 4.5. Trypsin–Bantam Interaction Analyses through Co-Expression and Target Prediction Analyses

The potential role of *trypsin* genes as targets for *bantam* miRNAs was evaluated through analyses of co-expression and target site prediction by sequence similarity and putative binding sites. Differentially expressed *trypsin* genes and *bantam* miRNAs were selected for both analyses. Pearson’s correlation among TPM values from both datasets, including all the samples exposed and non-exposed to each drug, was calculated in the R software [50]. Plots for correlation analyses were constructed using the Corrplot package [51], considering as significant those correlations with *p*-value < 0.01. *Trypsins* and *bantams* with significant negative correlations (*p*-value < 0.01 and correlation value < −0.95) were included in the CIRCOS plot as links considering their genome localization. Only negative correlations were included because it was expected that miRNAs that regulate transcripts show opposite trends in gene expression.

Target prediction analyses were calculated using default parameters using two open-source software packages: RNA22 [52] and RNAHybrid [53]. Potential target sites for *bantam* miRNAs were considered if ΔG < −15 and *p*-value < 0.05 in the algorithms from both programs.

### 4.6. Target Prediction Validation through Luciferase Assay

The 3′-UTR region identified for the *trypsin 2* mRNA sequence was used as a template for *bantam* binding site prediction validation because it had the best ΔG and p-values. The binding site prediction was validated by functional analysis by Dual-Luciferase**^®^** Reporter Assay System (Promega, Madison, WI, USA). Briefly, the target sequence of *trypsin 2* was cloned in a pmirGLO Dual-Luciferase miRNA Target Expression Vector (Promega, USA). HeLa cell lines (CellZion SpA, Nueva Providencia, Chile, HeLa (ATCC CCL-2)) were seeded at 30.000 cells by well. After 48 h, cells were co-transfected with Lipofectamine 3000 (Invitrogen^®^, Waltham, MA, USA) with the construction pmir-GLO-3UTR_*trypsin-2* and a *bantam* mimic (aga-bantam mirVana (Ambion, Waltham, MA, USA). The sequence used for the *bantam* mimic corresponded to the same sequence used for qPCR validation analysis—TCAGCGTTCACAATGATCTCA, which correspond to a *bantam-3* member. As a control group, HeLa cells were co-transfected with a pmir-GLO vector and *bantam* mimic. The luciferase activity was measured with the Dual-Luciferase**^®^** Reporter Assay System (Promega, USA) and normalized with Renilla. The assays were performed in triplicates. Statistical analysis was conducted through one-way ANOVA using the GraphPad Prism v9.0 software (GraphPad Software, Inc., San Diego, CA, USA).

## 5. Conclusions

The genes coding for *trypsin* and *chymotrypsin* and *bantam* miRNAs were identified throughout the genome of the sea louse *C. rogercresseyi*. Expression patterns in susceptible and resistant sea lice populations to azamethiphos, deltamethrin, and cypermethrin molecules, correlations of expression trends between *trypsin* genes and *bantam* miRNAs, and target prediction validation by the luciferase assay demonstrated a tight molecular interaction between *bantam* miRNAs and *trypsin* genes, especially the *trypsin-2* gene. The regulation of the expression of *trypsin* genes by a *bantam* suggests that this miRNA could be involved in the drug sensitivity of the sea louse *C. rogercresseyi*. Further studies will be needed to confirm the role of *bantam–trypsin* interactions on the emergence of the pharmacological resistance of these parasite species.

## Figures and Tables

**Table 1:** *Trypsin* genes identified in the *Caligus rogercresseyi* genome.

Gene Name	Chromosome	Length (bp)	Strand	Transcripts	Transcript Length
*Trypsin-1*	Chr2	5343	−	1	871
*Trypsin-3-like*	Chr5	5134	−	1	1330
*Serine protease 48-like*	Chr5	4377	+	7	508
*Serine protease 53-like*	Chr5	4377	+	7	508
*Trypsin-1-like*	Chr5	4377	+	7	508
*Trypsin-1-like*	Chr5	4377	+	7	508
*Serine protease 53-like*	Chr5	4377	+	7	508
*Protease serine 9-like*	Chr5	4377	+	7	508
*Chymotrypsin-2-like*	Chr5	4377	+	7	508
*Trypsin-2*	Chr6	7501	+	2	862
*Trypsin-7*	Chr6	7501	+	2	862
*Trypsin*	Chr6	1885	−	2	559
*Trypsin*	Chr6	1885	−	2	559
*Anionic trypsin-2*	Chr10	365	?	1	365
*Anionic trypsin-2*	Chr10	1397	+	1	1268
*Inter-alpha-trypsin H3*	Chr15	8195	+	2	2453
*Trypsin II-P29*	Chr18	5255	+	1	1290
*Inter-alpha-trypsin H4*	Chr19	4865	+	2	4522
*Inter-alpha-trypsin H4*	Chr19	4865	+	2	4522
*Inter-alpha-trypsin H3*	Chr19	329	−	4	240
*Inter-alpha-trypsin H4*	Chr19	329	−	4	240
*Inter-alpha-trypsin H5*	Chr19	329	−	4	240
*Inter-alpha-trypsin H6*	Chr19	329	−	4	240
*Trypsin-1*	Chr1	1208	+	1	1018
*Anionic trypsin-1*	Chr1	831	−	1	703
*Inter-alpha-trypsin H4*	Chr3	3430	?	1	3430
*Chymotrypsinogen B*	Chr5	334	?	1	334
*Chymotrypsin-like Elastase*	Chr5	315	?	1	315
*Chymotrypsin B*	Chr5	448	+	1	386
*Anionic trypsin-2-like*	Chr5	614	+	1	545
*Anionic trypsin-2-like*	Chr5	496	−	1	427
*Trypsin eta*	Chr5	807	?	2	593
*Trypsin eta*	Chr5	807	?	2	593
*Chymotrypsin-like*	Chr10	7596	−	1	985
*BPTI/Kunitz protein*	Chr10	445	+	1	385
*Trypsin-1*	Chr12	646	?	1	646
*trypsin IP1-like*	Chr12	668	?	1	668
*Trypsin-like*	Chr13	6442	+	1	840
*Trypsin-like*	Chr13	2764	−	2	345
*Trypsin-like*	Chr13	2764	−	2	345
*Trypsin epsilon*	Chr13	540	?	2	540
*Trypsin epsilon*	Chr13	540	?	2	540
*Trypsin 5G1*	Chr13	518	+	2	434
*Trypsin 5G1*	Chr13	518	+	2	434
*Trypsin-1*	Chr13	44817	−	2	833
*Trypsin-1*	Chr13	44817	−	2	833
*Trypsin-5*	Chr13	440	?	1	440
*Trypsin*	Chr13	1143	?	1	1143
*Trypsin-5*	Chr13	521	?	2	521
*Kallikrein-11*	Chr13	521	?	2	521
*Trypsin-5*	Chr13	729	?	1	729
*Trypsin-5*	Chr13	691	?	1	591
*Trypsin epsilon*	Chr13	560	?	1	560
*Ovochymase-2-like*	Chr15	9412	−	6	2904
*Ovochymase-2-like*	Chr15	9412	−	6	2904
*Ovochymase-2-like*	Chr15	9412	−	6	2904
*Ovochymase-2-like*	Chr15	9412	−	6	2904
*Ovochymase-2-like*	Chr15	9412	−	6	2904
*Ovochymase-2-like*	Chr15	9412	−	6	2904
*Trypsin-1*	Chr18	1467	+	1	427

**Table 2:** Primers and probes used for RT-qPCR expression analysis.

Gene	Direction	Sequence (5′-3′)	R (5′)	Q (3′)
*B-tubulin*	Forward	GGCTTCCAGCAACTACAACCG		
	Reverse	TTTCGTAAATAGGGTACCCGT		
	Probe	ACATGGCACTTGGGCCATGCG	HEX	BHQ1
*Trypsin 2*	Forward	CCAAGGTGACGGACCTTTGA		
	Reverse	AACGCTGTGACGAGGTTCAT		
	Probe	CCTAAAGGTTAGCCCCGAGG	FAM	BHQ1
*Trypsin 5*	Forward	TCCATGGTCCCAAGGCAAGTT		
	Reverse	CATTCGTTTGACCAAGGACCT		
	Probe	CGCCATCAATCCTCCGCCGCA	TAMRA	BHQ2
*Bantam*	Undirected	TCAGCGTTCACAATGATCTCA		
*miR-2796*	Undirected	GTGTCCCAATTATAATGTCCA		

## Data Availability

The assembled genome used for sequence mapping and annotation has been deposited to the NCBI Assembly with the accession number ASM1338718v1 (Genbank project accession number: PRJNA551027). The transcriptome (mRNA-seq and small-RNA-seq) sequences used to identify *trypsin* and *bantam* sequences are deposited in the Sequence Read Archive (SRA) of NCBI Genbank under the accession code PRJNA559846.

## Supplementary Materials

The following are available online at www.mdpi.com/article/10.3390/ncrna7040076/s1, Table S1: Mapped reads corresponding to bantam miRNAs on the *C. rogercresseyi* and annotation using two miRNAs databases.
